# Ultra-Broadband Metasurface Absorber Enabled by a Central-Bar-Coupled Split-Disk Dimer

**DOI:** 10.3390/bios16080439

**Published:** 2026-08-14

**Authors:** Carlotta Panciera, Giuseppe Brunetti, Caterina Ciminelli, Muhammad A. Butt

**Affiliations:** 1 Optoelectronics Laboratory, Department of Electrical and Information Engineering, Politecnico di Bari, Via Orabona 4, 70125 Bari, Italy; giuseppe.brunetti@poliba.it (G.B.); caterina.ciminelli@poliba.it (C.C.); 2 Institute of Microelectronics and Optoelectronics, Warsaw University of Technology, Koszykowa 75, 00-662 Warsaw, Poland

**Keywords:** metasurface absorber, high-Q resonance, refractive-index biosensing, hybrid metal–dielectric, near-infrared

## Abstract

A hybrid metasurface absorber (MSA) based on a central-bar-coupled split-disk dimer is proposed and numerically investigated for high-resolution refractive-index sensing in the near-infrared spectral region. The metasurface consists of silicon nitride dielectric resonators integrated with a gold plasmonic layer, enabling strong electromagnetic confinement, enhanced light–matter interaction, and ultra-narrow resonant features within the 1000–1400 nm wavelength range. The optimized structure supports multiple resonant modes under both *x*- and *y*-polarized excitation, producing sharp reflection dips with full-width-at-half-maximum values as low as 0.58 nm and quality factors reaching 2007. Refractive-index sensing performance was evaluated by varying the aqueous superstrate refractive index from 1.33 to 1.35, resulting in bulk sensitivities up to 860 nm/RIU under normal incidence. The angular response was further analyzed for incidence angles up to 5°, revealing polarization-dependent resonance splitting and the emergence of additional high-Q resonant branches under oblique excitation. Several angularly induced resonances exhibit narrower linewidths than those observed at normal incidence while preserving high refractive-index sensitivity up to 870 nm/RIU. Electric-field distributions confirm strong field localization near the dielectric boundaries and coupling regions, validating the hybrid resonant mechanism responsible for the enhanced spectral selectivity and sensing performance. The proposed MSA provides a promising platform for compact and ultrasensitive biosensing applications.

## 1. Introduction

Metasurfaces have emerged as an effective platform for manipulating electromagnetic waves at subwavelength scales, particularly in applications that require strong light–matter interaction near a surface [1,2]. By engineering arrays of resonant meta-atoms, these structures enable precise control over local electromagnetic fields while maintaining a compact and planar geometry [3]. Compared to bulk metamaterials, metasurfaces offer lower losses, simpler fabrication, and easier integration with sensing platforms. Their ability to confine electromagnetic energy in the near-field region makes them especially suitable for refractive index sensing, where small changes in the surrounding dielectric environment must be accurately detected [4,5]. In the context of biosensing, refractive-index detection represents one of the most widely exploited label-free optical transduction mechanisms, since biomolecular recognition events occurring at the sensor surface induce a local modification of the dielectric environment within the evanescent-field region [6,7]. In biosensing applications, biomolecular binding events occurring at the sensor surface locally modify the refractive index near the sensing interface [8]. As a result, biological recognition events can be transduced into measurable changes in the optical response, such as resonance wavelength shifts or intensity variations, enabling label-free detection of biomolecular interactions without the need for fluorescent, enzymatic, or radioactive labels [9].

MSAs are particularly advantageous in refractive index sensing due to their sharp resonant response and high field localization [10,11]. When designed to achieve impedance matching with free space, these structures exhibit near-perfect absorption at specific resonance frequencies, resulting in well-defined spectral features [12]. Any variation in the refractive index of the medium in contact with the metasurface alters the effective electromagnetic environment, leading to measurable shifts in the resonance position or changes in absorption intensity [13]. The sensitivity of such devices is closely related to the strength of the localized fields and the quality factor of the resonance, both of which can be engineered through careful design of the unit cell geometry and material composition [14,15]. However, conventional single-resonance absorbers often suffer from limited sensitivity range and narrow operational bandwidth [1].

Hybrid MSAs address these limitations by combining multiple resonant modes and material responses within a unified structure, enabling enhanced sensing performance [16,17,18]. The co-existence of electric and magnetic resonances, along with potential higher-order modes, allows for stronger field confinement and increased interaction volume with the analyte. This improves refractive-index sensitivity and enables multi-band detection capabilities [19]. Additionally, the integration of tunable materials such as graphene or phase-change compounds provides dynamic control over resonance characteristics, allowing for real-time optimization of sensing conditions [20,21]. These combined features make hybrid MSAs attractive for high-resolution refractive-index sensing in biochemical detection, medical diagnostics, and environmental monitoring [3,17,22,23]. Although previously reported hybrid metasurface sensors based on nanodisk, nanopillar, and patch-type resonators have demonstrated promising refractive-index sensing performance, many existing designs remain constrained by relatively simple resonator geometries that provide limited flexibility for engineering multiple spectrally selective resonant modes [24,25,26]. Simultaneously achieving narrow linewidth, strong field confinement, and polarization- or angle-dependent tunability within a compact hybrid metasurface platform therefore remains a significant challenge, particularly in the near-infrared spectral region [27].

To address these limitations, this work proposes a hybrid MSA based on a central-bar-coupled split-disk dimer, designed to exploit the complementary advantages of dielectric and plasmonic constituents. Unlike previously reported nanodisk- [24], nanopillar- [25], or patch-based hybrid absorbers [26], the proposed geometry is designed to integrate the two Si_3_N_4_ semicylindrical sections, the central connecting bar, and the underlying Au layer within the same compact unit cell. In this configuration, all three elements directly contribute to the localization of the electromagnetic field and to the formation of multiple resonances exploited for refractive index biosensing. This design therefore aims to improve the balance between spectral sharpness, refractive-index sensitivity, and multi-channel sensing capability while preserving a relatively simple meta-atom layout. Furthermore, the influence of polarization and small angular deviations from normal incidence is systematically examined to assess the stability and evolution of the resonant response. The study combines geometrical optimization, electromagnetic modeling, and field analysis to provide physical insight into the resonant mechanisms governing the sensing behavior of the proposed metasurface platform.

## 2. Design Methodology and Computational Modeling

**Figure 1:**
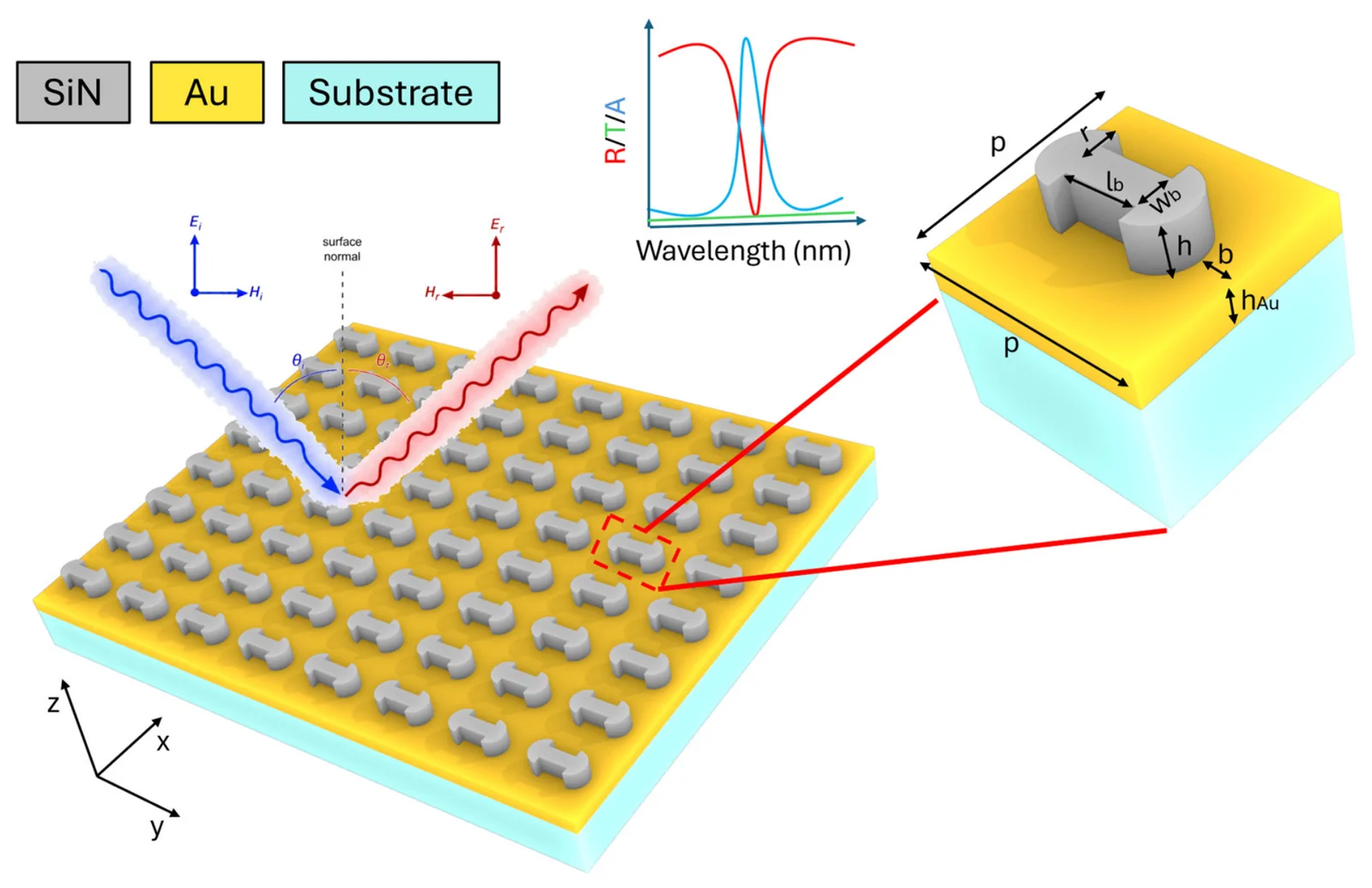
Schematic of the MSA and its optical response. A periodic array of silicon nitride (Si_3_N_4_) nanostructures on a gold (Au) film over a substrate is illuminated by an incident wave, producing a reflected wave. The inset shows the spectral response, and the zoomed view defines the unit-cell geometry. Blue is the incident light, and red is the reflected light.

The proposed structure consists of a hybrid metasurface, schematically illustrated in Figure 1. The metasurface is periodic along both the *x*- and *y*-directions and is excited from the top side (*z*-direction) under a nearly normal angle of incidence (AOI). The unit cell is composed of a silica substrate (SiO_2_, n ≈ 1.45), a continuous gold layer acting as the plasmonic component, and a dielectric meta-atom made of silicon nitride (Si_3_N_4_, n ≈ 2). The meta-atom consists of two semicylindrical elements connected by a central bar, forming the elementary resonant building block periodically repeated over the surface.

An aqueous superstrate (n ≈ 1.33) was considered to reproduce the biosensing environment. The analyte was then assumed to be contained in this upper aqueous superstrate, placed above and in direct contact with the exposed metasurface; in the subsequent sensing analysis, its presence was modeled by varying the superstrate refractive index from 1.33 to 1.35. In the present model, the aqueous medium was treated as a lossless dielectric with a constant refractive index, and wavelength-dependent absorption losses of water within the investigated near-infrared range were not explicitly included. This assumption was adopted to isolate and evaluate the intrinsic resonant and refractive-index sensing behavior of the proposed metasurface. Nevertheless, the considered spectral window remains below the stronger water absorption bands occurring at longer near-infrared wavelengths, suggesting that the general sensing trends and resonant behavior are expected to remain qualitatively preserved. The sensing mechanism relies on the hybrid nature of the structure: the gold layer is exploited to enhance the evanescent electromagnetic field at the metal–dielectric–aqueous interface, where biomolecular binding events are expected to occur, thereby improving refractive-index sensitivity. At the same time, the Si_3_N_4_ dielectric resonator is introduced to reduce optical losses and support high-Q resonant features, leading to a narrow reflection dip suitable for accurate spectral read-out.

The structure was investigated in the wavelength range of 1000 nm–1400 nm because this near-infrared region is highly suitable for sensing owing to its low loss and enhanced optical sensitivity. To obtain a resonant response within the target wavelength range and to enhance the spectral performance of the device, the main geometrical parameters are defined as follows: the gold-layer thickness is set to h_Au_ = 100 nm, while the Si_3_N_4_ meta-atom thickness is h = 150 nm. The two semi-cylindrical elements have radius r = 200 nm and are connected by a central bar with length l_b_ = 175 nm and width w_b_ = 100 nm. The period is first defined along the *y*-direction as p = 2r + l_b_ + 2b, where b is the distance between the end of the dielectric structure and the boundary of the unit cell along *y*. In the optimized configuration, b = 200 nm, resulting in p = 975 nm. Once p is defined, the basic unit cell is chosen to be square, with dimensions pxp as shown in the inset of Figure 1. Both linear polarization states were considered by orienting the electric field of the incident electromagnetic wave along either the *x*- or *y*-direction. Although the main operating condition corresponds to normal incidence, with an AOI = 0°, the angular response was also tested up to ±5° to evaluate the resonant behavior with respect to small deviations from normal excitation.

The electromagnetic response of the proposed metasurface sensor was numerically investigated using a three-dimensional Finite Element Method (FEM) model implemented in COMSOL Multiphysics version 6.4 (COMSOL, Stockholm, Sweden). Material dispersion was taken into account through the COMSOL Optical Material Library, using the “Au (Johnson and Christy 1972: n,k 0.188–1.937 µm)” dataset for the gold layer and the “Si_3_N_4_ (Silicon Nitride)—Luke” dataset for the dielectric resonator. Floquet periodic boundary conditions were imposed on the lateral faces of the unit cell to represent the infinite periodicity of the metasurface in the *x*- and *y*-directions. To minimize non-physical reflections and emulate an open-domain environment, perfectly matched layers (PMLs) were applied above the aqueous sensing medium and below the substrate. The structure was illuminated by a normally incident plane wave propagating along the *z*-axis, and the optical response was analyzed within the near-infrared wavelength range of 1000 nm–1400 nm using a wavelength step of 0.5 nm.

## 3. Parametric Optimization

**Figure 2:**
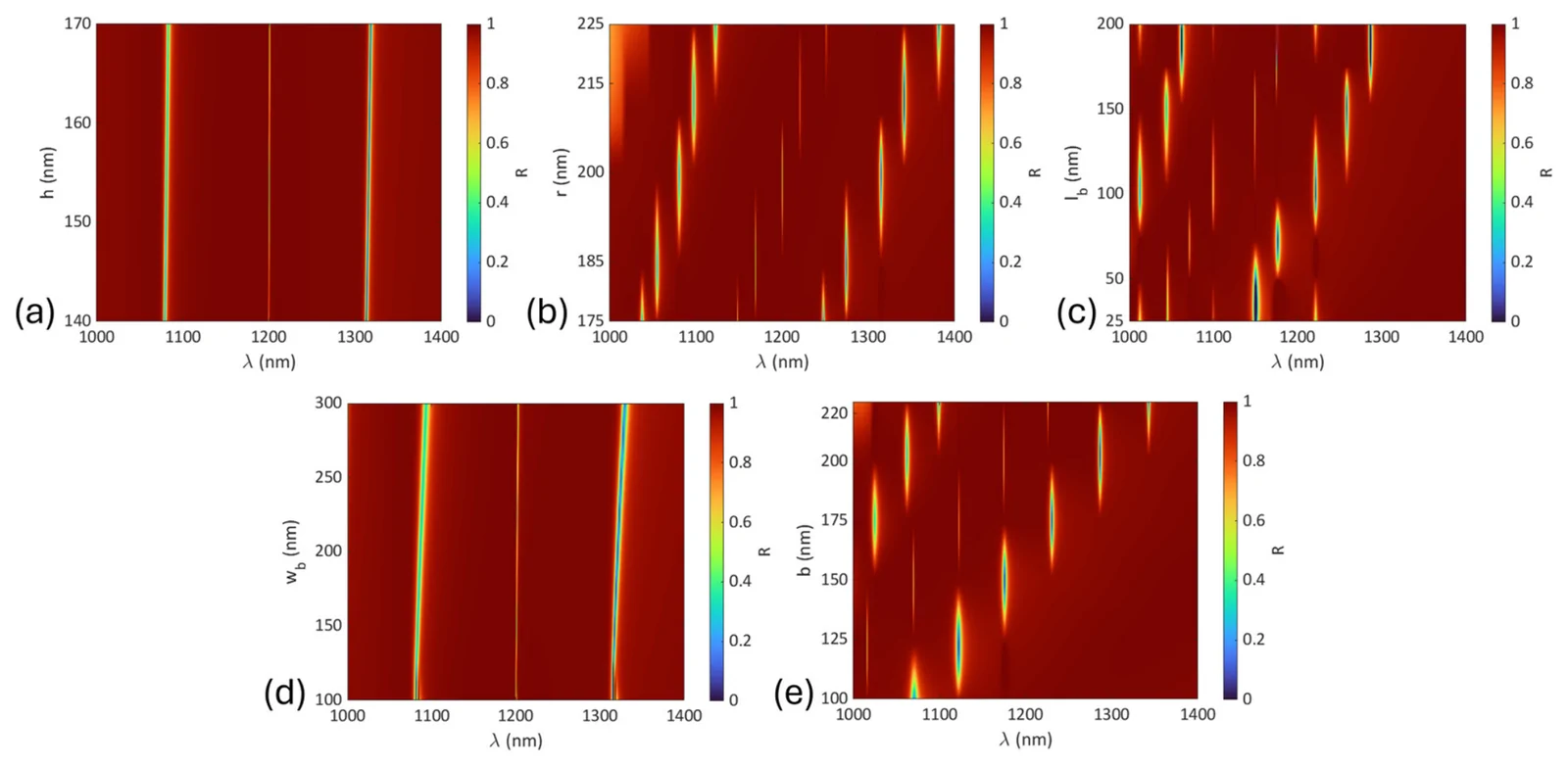
Reflection (R) colormaps as a function of wavelength (λ) and the swept geometrical parameter. Each map was obtained by varying one parameter at a time while keeping all the others fixed. Specifically, the varied parameter is (**a**) the structure height (h); (**b**) the radius of the semicylindrical element (r); (**c**) the length of the central bar (l_b_); (**d**) the width of the central bar (w_b_); and (**e**) the parameter (b), defined as the distance between the dielectric structure and the unit-cell boundary along the *y*-direction.

The optimization of the geometrical parameters was carried out under *x*-oriented electric-field polarization by evaluating each dimension independently, keeping all the other parameters fixed while varying one at a time. A sequential parametric sweep approach was adopted because the number of design variables remained relatively limited and each parameter exerts a physically distinct influence on the resonant characteristics of the metasurface. This methodology allows for direct interpretation of the individual role of each geometrical parameter in controlling resonance position, linewidth, and reflection depth. Because the metasurface topology and target spectral range were predefined, each parameter was varied over a broad interval to compare several feasible configurations and identify the best balance among resonance position, linewidth, and reflection depth. Although it does not fully capture the strong coupling that can exist among geometrical parameters or establish global optimality, it provides a consistent and physically grounded optimization within the investigated design space. The first parameter to be optimized was the Si_3_N_4_ meta-atom height, h. It was varied from 140 nm to 170 nm while keeping the other geometrical parameters fixed (w_b_ = 100 nm, l_b_ = 200 nm, r = 200 nm and p = 1000 nm). As shown in Figure 2a, varying the Si_3_N_4_ height h directly modifies the vertical modal confinement within the dielectric meta-atom, producing clear changes in the resonance depth and spectral definition; among the investigated values, h = 150 nm provides the most well-defined resonant dips within the target wavelength range.

With the optimized height fixed at 150 nm, the radius r was varied from 175 nm to 225 nm, while the unit-cell period was adjusted according to p = 2r + l_b_ + 2b. Figure 2b shows that varying r produces a continuous shift in the resonant features together with changes in dip contrast, indicating that the lateral size of the semi-cylindrical sections controls the effective optical path and the hybrid modal confinement without suppressing the resonant response within the investigated range. The relatively continuous evolution of the resonant features observed over the investigated radius range also suggests that moderate fabrication-induced dimensional deviations in this parameter are not expected to suppress the fundamental resonant behavior of the metasurface. Accordingly, r = 200 nm was selected because it provides well-defined resonant dips with a favorable balance between spectral position, linewidth, and resonance depth. As the third optimization step, the length of the central connecting bar (l_b_) was investigated to evaluate its influence on the resonant coupling between the two semi-cylindrical dielectric elements. The parameter l_b_ was varied from 25 nm to 200 nm. The bar length directly influences the electromagnetic interaction between the two dielectric sections by modifying the effective coupling region and the distribution of the confined fields within the meta-atom. The trend in Figure 2c shows that l_b_ is mainly responsible for tuning the intra-dimer coupling: different bar lengths modify the hybridization between the two semi-cylindrical sections, producing changes in resonance position and dip contrast. The selected value l_b_ = 175 nm yields clearly distinguishable resonant features, which is essential for reliable extraction of linewidth and sensing metrics.

As the fourth optimization step, the width of the central bar (w_b_) was varied between 100 nm and 300 nm. Since the bar width w_b_ controls the effective coupling area between the two dielectric sections, it strongly affects both local field confinement and resonance sharpness. As shown in Figure 2d, increasing w_b_ weakens the spectral definition of the resonant dips, indicating that excessive widening of the central coupling region is detrimental to narrow-linewidth operation. Because this parameter directly controls the electromagnetic coupling strength between the two dielectric sections, it also represents one of the most fabrication-sensitive dimensions, and small deviations may lead to measurable resonance shifts and linewidth variations. The smallest investigated value, w_b_ = 100 nm, provides the most pronounced and well-defined resonant response and was therefore selected as the optimized value.

Finally, a parametric sweep of b, defined as the distance between the dielectric structure and the unit-cell boundary along the *y*-direction, was carried out to determine the optimal periodic spacing of the metasurface. All previously optimized geometrical parameters were fixed, while b was varied from 100 nm to 220 nm. Since b directly affects the separation between adjacent unit cells, it influences near-field coupling and the collective resonant behavior of the periodic metasurface array. The trend in Figure 2e confirms that the periodic spacing controls the strength of inter-cell coupling: smaller values increase interaction between neighboring unit cells, whereas larger values progressively reduce collective coupling effects. Based on this analysis, the optimized value was selected as b = 200 nm, resulting in a final unit-cell period of p = 975 nm, thereby completing the geometrical optimization of the proposed metasurface structure. Therefore, after completion of the structural optimization process, the final geometrical parameters were defined as h = 150 nm, r = 200 nm, l_b_ = 175 nm, w_b_ = 100 nm, and b = 200 nm, which correspond to a final unit-cell period of p = 975 nm.

## 4. Performance Analysis

Following the geometrical optimization, the performance of the proposed MSA was systematically evaluated to assess its resonant characteristics and refractive-index sensing capability. The analysis includes the optical response under normal-incidence excitation, the influence of small angular deviations from normal incidence, and the corresponding electric-field distributions to clarify the underlying resonant mechanisms governing the observed spectral behavior.

### 4.1. Sensing Performance with 0° AOI

After the optimization procedure, the final metasurface configuration was analyzed under plane-wave excitation by considering different orientations of the electric-field component of the incident electromagnetic wave. In particular, the optical response of the structure was evaluated for both *y*- and *x*-polarized excitation conditions. The corresponding reflection spectrum exhibits three distinct resonant dips within the investigated spectral range. The physical origin of these reflectance dips can be attributed to hybrid resonant interactions arising from the combined dielectric and plasmonic characteristics of the proposed metasurface. The Si_3_N_4_ split-disk dimer supports localized dielectric resonances associated with displacement-current oscillations and strong electromagnetic confinement within the high-index dielectric structure. Simultaneously, the underlying Au layer enhances electromagnetic confinement near the metal–dielectric interface by increasing local field concentration and strengthening light–matter interaction. The interaction between these dielectrics and plasmonic responses, together with electromagnetic coupling introduced by the central connecting bar and the periodic arrangement of neighboring unit cells, generates multiple spectrally selective hybrid resonant modes.

First, the case corresponding to the electric field oriented along the *y*-direction is considered. Under this excitation condition, the metasurface supports three well-defined resonances located at λ_1_ = 1070 nm, λ_2_ = 1175.5 nm, and λ_3_ = 1310, respectively. To characterize the spectral response of the device, the full width at half maximum (FWHM) of each resonance was evaluated. The extracted FWHM values are 1.93 nm for the first dip (1), 1.19 nm for the second dip (2), and 1.84 nm for the third dip (3).

The quality factor (*Q*) of each resonance was also calculated according to the relation *Q* = λ_R_/FWHM, where λ_R_ denotes the resonance wavelength [28]. The extracted *Q* values are 555.35, 990.66, and 709.80 for the first, second, and third resonances, respectively. Among the observed modes, the second resonance exhibits the highest *Q*-factor, arising from its substantially narrower linewidth compared with the other resonances.

**Figure 3:**
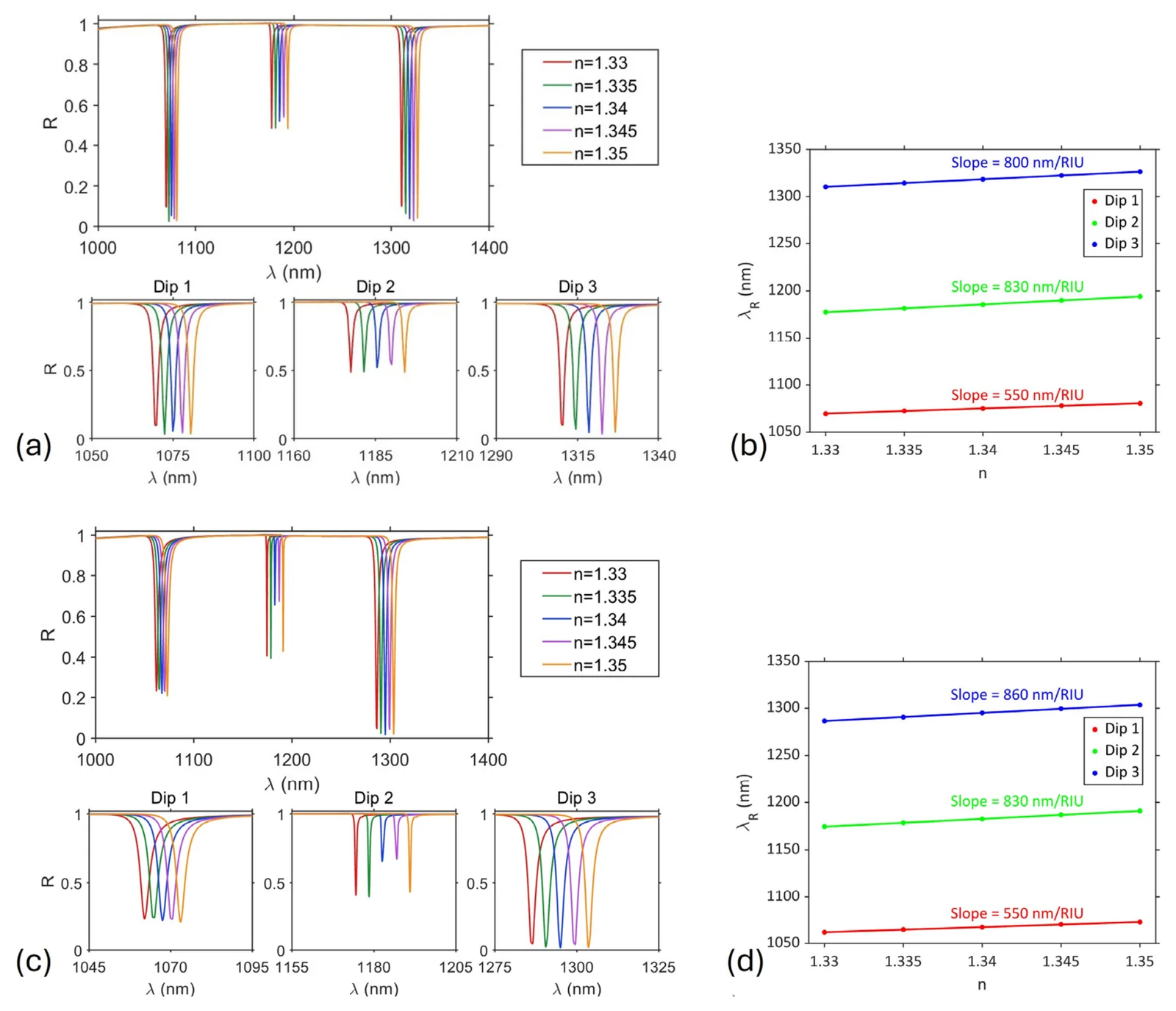
Reflection spectra for different refractive indices of the aqueous superstrate (n = 1.33–1.35): (**a**) *y*-polarized excitation and (**c**) *x*-polarized excitation. Corresponding bulk-sensitivity analyses are shown in (**b**,**d**).

Subsequently, the sensing performance of the MSA was evaluated by varying the refractive index of the aqueous superstrate. Starting from the reference condition n = 1.33, corresponding to the absence of analytes and represented by the red spectrum in Figure 3a, the superstrate refractive index was progressively increased up to n = 1.35. This homogeneous refractive-index variation was adopted as a first-step numerical assessment of the response of the resonant modes to changes in the surrounding dielectric environment, such as those potentially associated with an increasing concentration of a target analyte in the sensing medium [5].

The corresponding reflection spectra are reported in Figure 3a, showing a clear spectral shift in the three resonant dips as the refractive index of the superstrate increases. The bulk sensitivity S_B_, defined as the resonance wavelength shift per refractive-index unit S_B_ = Δλ\Δn [5], was then extracted for each resonance. To further evaluate the sensing performance by simultaneously considering both resonance sensitivity and spectral linewidth, the figure of merit (FOM) was calculated according to the relation FOM = S_B_/FWHM, where S_B_ represents the bulk sensitivity and FWHM denotes the full width at half maximum of the corresponding resonance. As shown in Figure 3b, the three dips exhibit sensitivity values of 550 nm/RIU, 830 nm/RIU, and 800 nm/RIU, respectively. Dip 2 provides the highest sensitivity among the three resonances while simultaneously exhibiting the narrowest linewidth and highest Q-factor, resulting in the highest FOM. Compared with Dip 1 and Dip 3, the narrower linewidth of Dip 2 reduces spectral uncertainty and enables improved wavelength discrimination during spectral interrogation. This indicates that sensing performance is not governed solely by resonance shift, but also by resonance linewidth, since the combination of high sensitivity and enhanced spectral confinement allows for more accurate detection of small refractive-index perturbations.

The analysis was then extended to the case in which the incident electric field is polarized along the *x*-direction. Following the same procedure adopted for the *y*-polarized excitation, the optical response of the metasurface was first evaluated.

Also in this configuration, the reflection spectrum exhibits three distinct resonant dips located at λ_1_ = 1070 nm, λ_2_ = 1175.5 nm, and λ_3_ = 1310. The extracted FWHM values are 3.42 nm, 0.58 nm, and 2.78 nm for the first, second, and third resonances, respectively. Using the same definition previously introduced, the corresponding *Q*-factors were found to be 310.78, 2007.01, and 463.17. In particular, the second resonance exhibits an extremely narrow linewidth, resulting in a remarkably high *Q*-factor exceeding 2000, indicative of very strong spectral confinement and enhanced spectral selectivity. For particular resonant modes, the observed reduction in resonance linewidth may be associated with interference between resonant pathways exhibiting different radiative efficiencies, resulting in reduced radiative damping and an enhanced Q-factor. The corresponding spectral behavior is qualitatively consistent with Fano-like interference commonly observed in coupled metasurface resonators.

The sensing performance was subsequently evaluated by repeating the refractive-index analysis described for the *y*-polarized excitation. As reported in Figure 3c,d, the obtained sensitivity values are 550 nm/RIU, 830 nm/RIU, and 860 nm/RIU for the first, second, and third resonances, respectively. Although the resonance positions under *x*- and *y*-polarized excitation appear close, the spectra are not identical; clear differences are observed in resonance linewidth, dip depth, Q-factor, sensitivity, and electric-field localization, confirming polarization-dependent coupling to the hybrid resonant modes.

To complement the bulk refractive-index analysis and more realistically reproduce a surface-binding biosensing configuration, an additional set of simulations was performed by introducing a biomolecular layer over the entire exposed surface of the Si_3_N_4_ meta-atom. The layer was assigned a thickness of h_b_ = 10 nm and a refractive index of n_b_ = 1.45, representing an effective anti-IgG/IgG biofunctionalization and binding layer formed on the sensor surface [5]. This finite-thickness model accounts for the fact that, in a practical immunosensing configuration, the refractive-index perturbation is localized within a nanoscale region surrounding the resonator rather than distributed throughout the entire aqueous superstrate. The model assumes uniform and complete surface coverage and does not explicitly account for binding efficiency, non-uniform coverage, or analyte-specific surface interactions.

The reflection spectra calculated before and after the introduction of the biomolecular layer were compared for both *y*- and *x*-polarized excitation. The surface sensitivity (S_S_) was expressed here as the resonance-wavelength shift per unit thickness of the biomolecular layer: S_S_ = Δλ_R_/Δh_b_ (pm/nm) [29]. For both electric-field orientations, surface-sensitivity values of approximately 150 pm/nm, 50 pm/nm, and 200 pm/nm were obtained for Dip 1, Dip 2, and Dip 3, respectively. Among the three modes, Dip 3 exhibits the largest surface sensitivity, whereas Dip 2, despite showing the highest Q-factor and bulk sensitivity, provides the lowest thickness-normalized response to the biomolecular layer.

### 4.2. Sensing Performance with Different AOI

The angular response of the proposed MSA was then investigated by varying AOI from normal incidence up to 5° to evaluate the stability and evolution of the resonant response under oblique excitation. When the AOI deviates from 0°, the incident electromagnetic wave acquires a finite in-plane wave-vector component, which modifies the excitation conditions and perturbs the symmetry of the incident field distribution. Consequently, resonant states that are spectrally overlapped or weakly excited at normal incidence can become distinguishable under oblique illumination, leading to resonance splitting and angular dispersion of the spectral response.

Under normal incidence, the excitation symmetry restricts coupling to specific hybrid electromagnetic states formed through the interaction of localized dielectric resonances within the Si_3_N_4_ structure, field confinement near the Au interface, and near-field interactions mediated by the periodic lattice. As the angle of incidence increases, the additional in-plane momentum alters the phase-matching conditions and breaks the symmetry constraints. This enables independent excitation of previously degenerate or partially overlapped hybrid modes, causing them to separate into distinct resonant branches. The resulting angular dispersion therefore reflects continuous redistribution of the electromagnetic coupling strength among these interacting resonant states.

**Figure 4:**
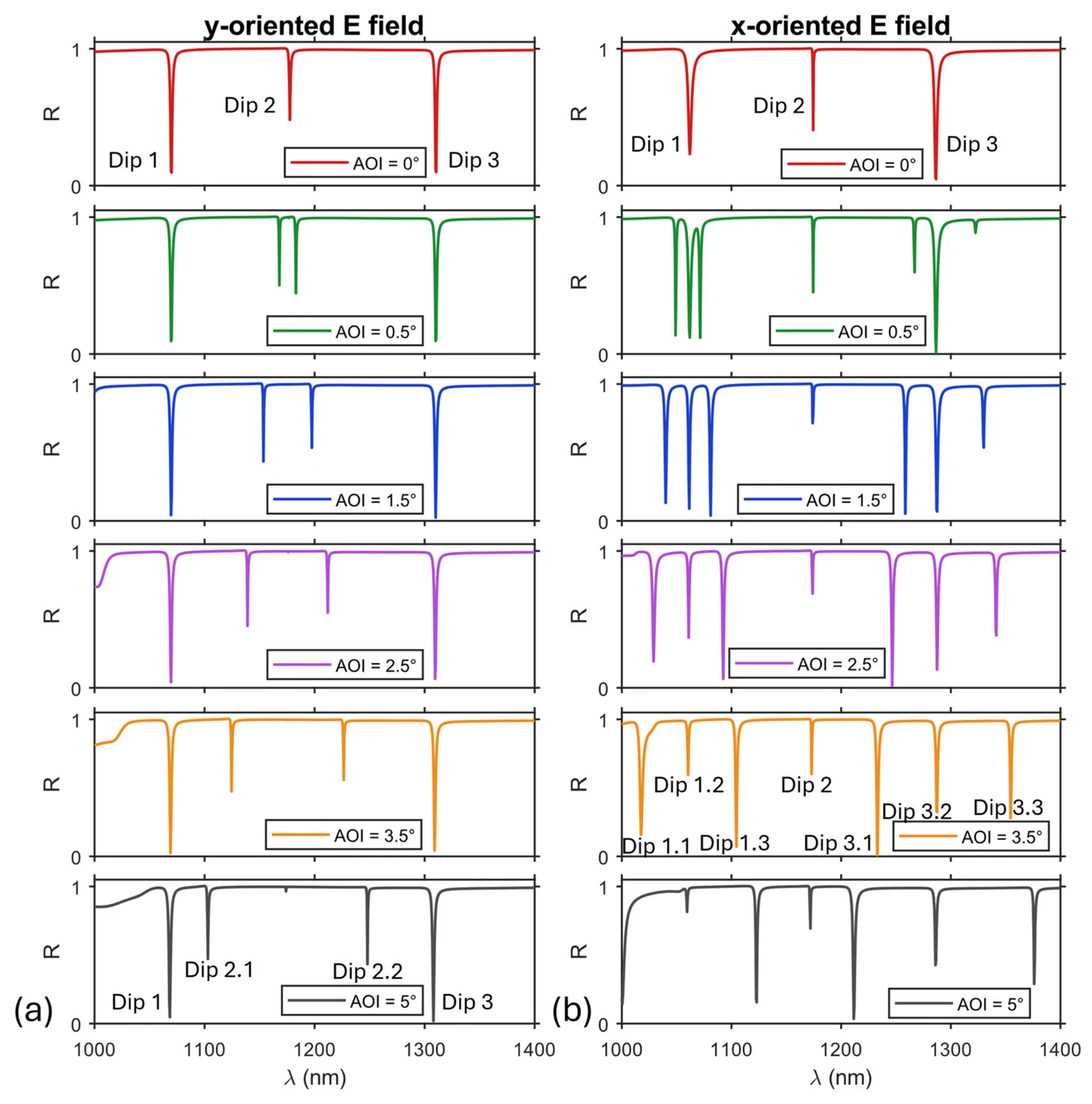
Reflection spectra of the metasurface for different AOI, ranging from normal incidence 0° up to 5°. (**a**) *y*-Oriented electric field and (**b**) *x*-oriented electric field. Each row corresponds to a progressively increasing incidence angle, specifically AOI = 0°, 0.5°, 1.5°, 2.5°, 3.5°, and 5°, respectively.

For *y*-oriented electric-field excitation (Figure 4a), the overall spectral response remains relatively stable over the investigated angular range, with the main angular effect occurring for Dip 2. At normal incidence, this mode appears as a single resonance dip; however, under oblique excitation, it progressively splits into two distinct resonant branches. This behavior suggests that the original resonance may originate from two closely spaced resonant states that remain spectrally overlapped at AOI = 0° because of the symmetry of the excitation. The introduction of a nonzero in-plane momentum component enables independent coupling to these resonant states, resulting in spectral splitting. The split resonances also exhibit reduced linewidths compared with the original resonance at normal incidence. Specifically, while the central dip at AOI = 0° exhibits an FWHM of 1.19 nm, the split resonances under oblique excitation show narrower linewidths ranging from approximately 0.64 nm to 0.86 nm. This linewidth reduction suggests that oblique excitation modifies the radiative coupling of the resonant states and partially suppresses radiative broadening, leading to enhanced spectral confinement.

A substantially different angular behavior is observed for *x*-oriented electric-field excitation (Figure 4b). In this case, Dip 2 remains comparatively stable, whereas the two lateral resonances exhibit pronounced angular splitting. As the AOI increases, each lateral dip evolves into three distinct resonant branches, indicating stronger angular sensitivity and more complex resonant interactions for modes excited under *x*-oriented electric-field illumination. This behavior can be attributed to the anisotropic geometry of the central-bar-coupled split-disk dimer and to the stronger overlap between the *x*-oriented electric field and the asymmetric current distributions supported by the central connecting bar and curved dielectric sections. Under oblique incidence, the additional in-plane momentum enhances the coupling among resonant interactions associated with the dielectric resonator, the Au layer, and the periodic arrangement of the metasurface. Consequently, the original resonances separate into multiple spectrally resolved branches.

The linewidth evolution under *x*-oriented electric-field excitation further confirms the formation of sharper resonant states under oblique incidence. While the two outer resonances at normal incidence exhibit FWHM values of 3.42 nm and 2.78 nm, respectively, the split resonances generated at nonzero AOI generally present narrower linewidths ranging from approximately 0.9 nm to 2.9 nm. The narrower spectral features indicate reduced radiative damping and enhanced modal selectivity for several of the angularly induced resonances. Overall, the angular response demonstrates that the metasurface preserves well-defined high-Q resonant characteristics under small angular perturbations, while simultaneously supporting additional angle-dependent resonant channels arising from symmetry perturbation and polarization-dependent resonant coupling.

**Figure 5:**
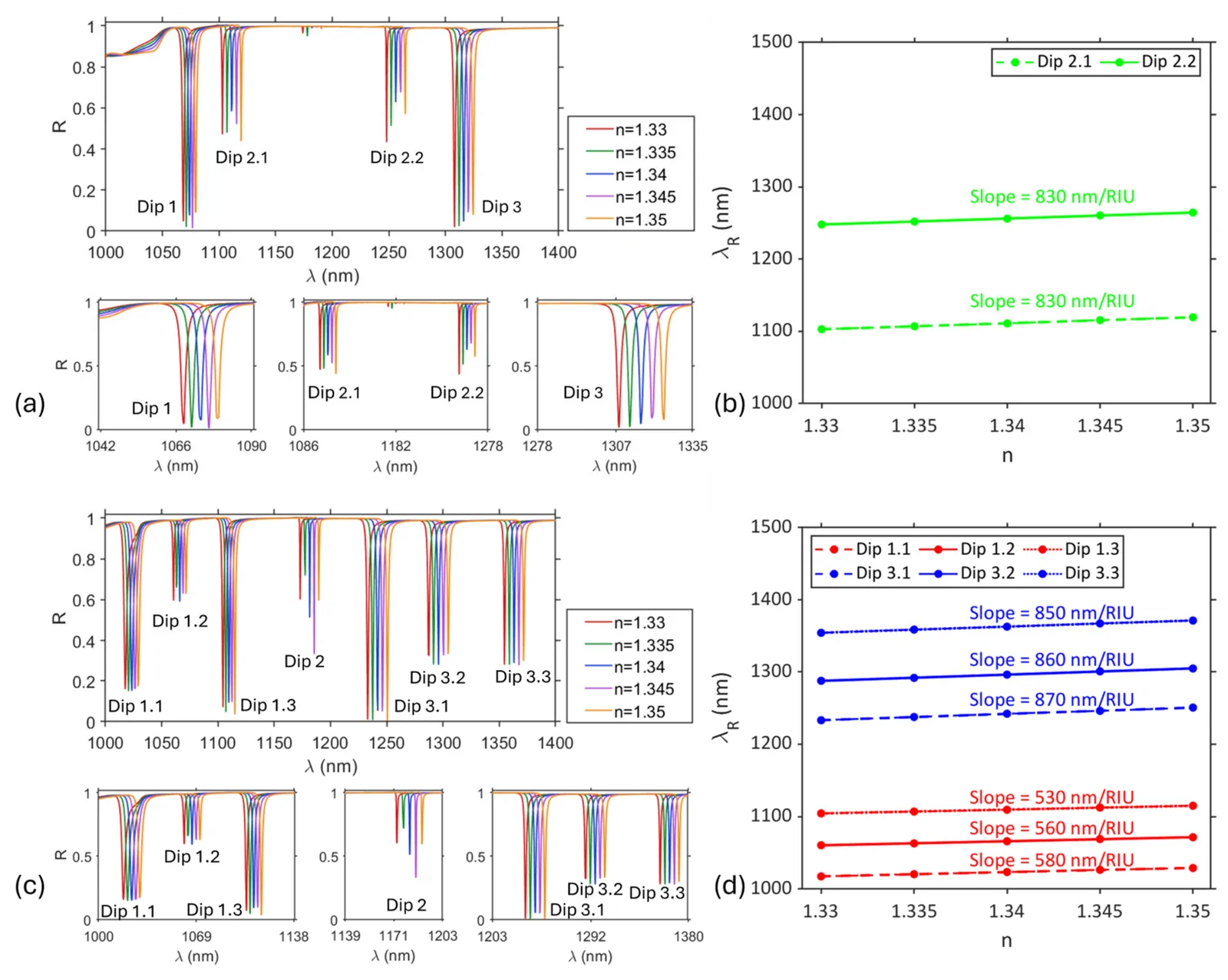
Biosensing performance of the metasurface under oblique-incidence excitation. (**a**) Reflection spectra obtained for *y*-polarized excitation at AOI = 5° by varying the refractive index of the aqueous superstrate from n = 1.33 to n = 1.35. (**b**) Corresponding resonance wavelength shifts and extracted bulk sensitivity values for the two resonant branches generated by the splitting of the central dip under *y*-polarized excitation. (**c**) Reflection spectra obtained for *x*-polarized excitation at AOI = 3.5° for different superstrate refractive-index values. (**d**) Corresponding resonance wavelength shifts and bulk sensitivity values for the six resonant branches originating from the splitting of the two lateral resonances under *x*-polarized excitation.

To further investigate the sensing capabilities of the proposed metasurface under oblique excitation, an additional refractive-index analysis was carried out by considering nonzero angles of incidence. In particular, the analysis was performed at AOI = 5° for the *y*-polarized excitation and at AOI = 3.5° for the *x*-polarized excitation. Different incidence angles were selected for the two polarization states to maintain all the resonant features within the investigated spectral window. Indeed, for the *x*-polarized configuration, the progressive splitting of the lateral resonances caused some of the resonant branches to move outside the analyzed wavelength range when the incidence angle approached 5°. Also in this case, the refractive index of the aqueous superstrate was varied from n = 1.33, corresponding to the reference sensing condition, up to n = 1.35. The resulting shifts in the resonance wavelengths (Figure 5a,c) were then used to evaluate the bulk sensitivity of the different resonant branches.

For the *y*-polarized excitation, the oblique incidence causes the original Dip 2 to split into two distinct dips. Therefore, the sensitivity analysis was specifically performed on these two resonant branches, as reported in Figure 5b. Both split resonances exhibit a bulk sensitivity of approximately 830 nm/RIU.

For the *x*-polarized excitation, the sensing analysis was extended to the six resonant branches generated by the splitting of the two lateral dips. As previously discussed, the two outer resonances split into three distinct resonant branches each, resulting in a total of six resonant dips. The sensitivity was therefore evaluated independently for all these resonances. The extracted values, reported in Figure 5d, are equal to 580 nm/RIU, 560 nm/RIU, and 530 nm/RIU for the three resonances originating from the first lateral dip, and 870 nm/RIU, 860 nm/RIU, and 850 nm/RIU for the three resonances associated with the second lateral dip. The main results discussed are summarized in Table 1 for performance comparison.

### 4.3. Electric-Field Distribution Analysis

**Figure 6:**
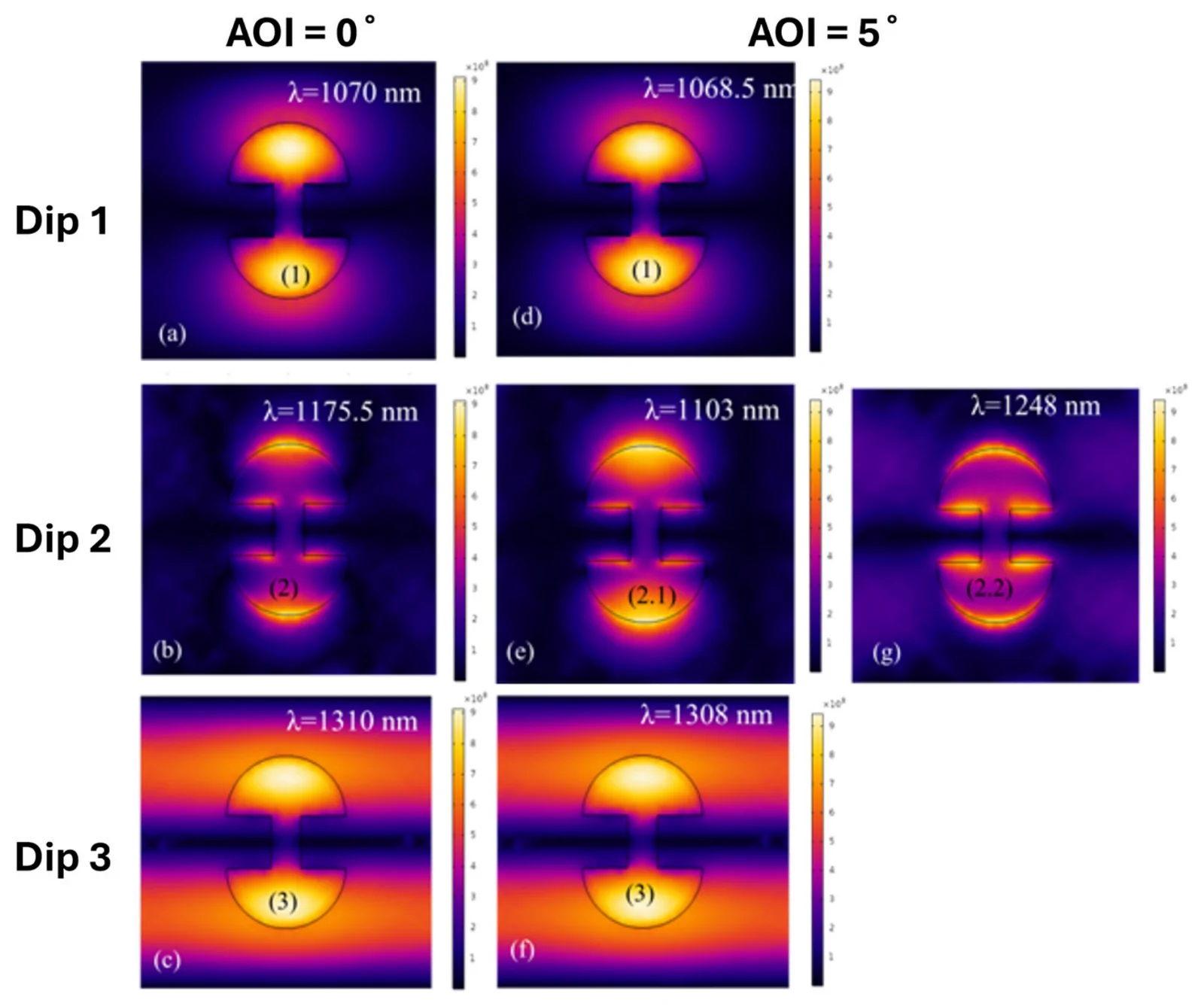
Electric-field distributions illustrating resonance evolution under *y*-oriented electric-field excitation: (**a**–**c**) correspond to Dip 1, Dip 2, and Dip 3 under normal incidence (AOI = 0°); (**d**–**g**) show the corresponding resonance evolution at AOI = 5°, where Dip 1 and Dip 3 remain largely unchanged, while Dip 2 splits into two distinct resonant branches (Dip 2.1 and Dip 2.2).

The electric-field distributions shown in Figure 6 provide physical insight into the hybrid resonant mechanisms responsible for the reflectance dips observed in the optical spectra under *y*-oriented electric-field excitation. Detailed information can be found in Supplementary Note S1. The field maps reveal distinct spatial localization patterns, suggesting that the different spectral dips are associated with distinct resonant field distributions and coupling conditions. In particular, variations in field localization within the dielectric boundaries, central coupling region, and metal–dielectric interface suggest that each resonance is associated with a distinct confinement state and coupling strength, which directly influences the observed differences in linewidth, Q-factor, and refractive-index sensitivity. In all cases, strong field enhancement is observed near the dielectric resonator boundaries and around the central connecting region, confirming efficient electromagnetic energy localization within the metasurface unit cell.

At normal incidence (AOI = 0°), the resonance at λ = 1070 nm (Figure 6a) exhibits strong field localization within the upper and lower semicircular sections of the resonator, with a relatively symmetric field distribution. For the resonance at λ = 1175.5 nm (Figure 6b), the electric field becomes more strongly confined near the curved dielectric edges and the junctions connecting the central bar to the semicircular sections. This stronger localization is consistent with the narrower linewidth and higher *Q*-factor observed for this resonance. In contrast, the resonance at λ = 1310 nm (Figure 6c) exhibits a broader field distribution extending further into the surrounding medium, indicating stronger interaction between the evanescent field and the external sensing environment.

Under oblique excitation (AOI = 5°), the field distributions directly reveal the resonance evolution observed in Figure 4a. Dip 1 shifts slightly from 1070 nm to 1068.5 nm, with Figure 6a,d showing nearly identical field distributions, indicating that this mode undergoes only weak angular perturbation. A similar behavior is observed for Dip 3, where Figure 6c,f exhibit comparable field distributions despite the small spectral shift from 1310 nm to 1308 nm, explaining the relatively stable spectral behavior of this resonance branch. The strongest angular effect occurs for Dip 2. Under normal incidence, Dip 2 (Figure 6b) exhibits strong field confinement near the inner dielectric boundaries and central coupling region. Under oblique excitation, symmetry breaking caused by the finite in-plane wave vector leads to splitting of this resonance into two distinct branches at λ = 1103 nm and λ = 1248 nm, shown in Figure 6e,g, respectively. Compared with the original mode, Dip 2.1 exhibits stronger localization near the central coupling region, whereas Dip 2.2 shows enhanced field concentration near the outer curved dielectric boundaries. This redistribution of electromagnetic confinement indicates modified modal coupling conditions under oblique excitation and explains the resonance splitting and formation of spectrally distinct branches observed in Figure 4a.

**Figure 7:**
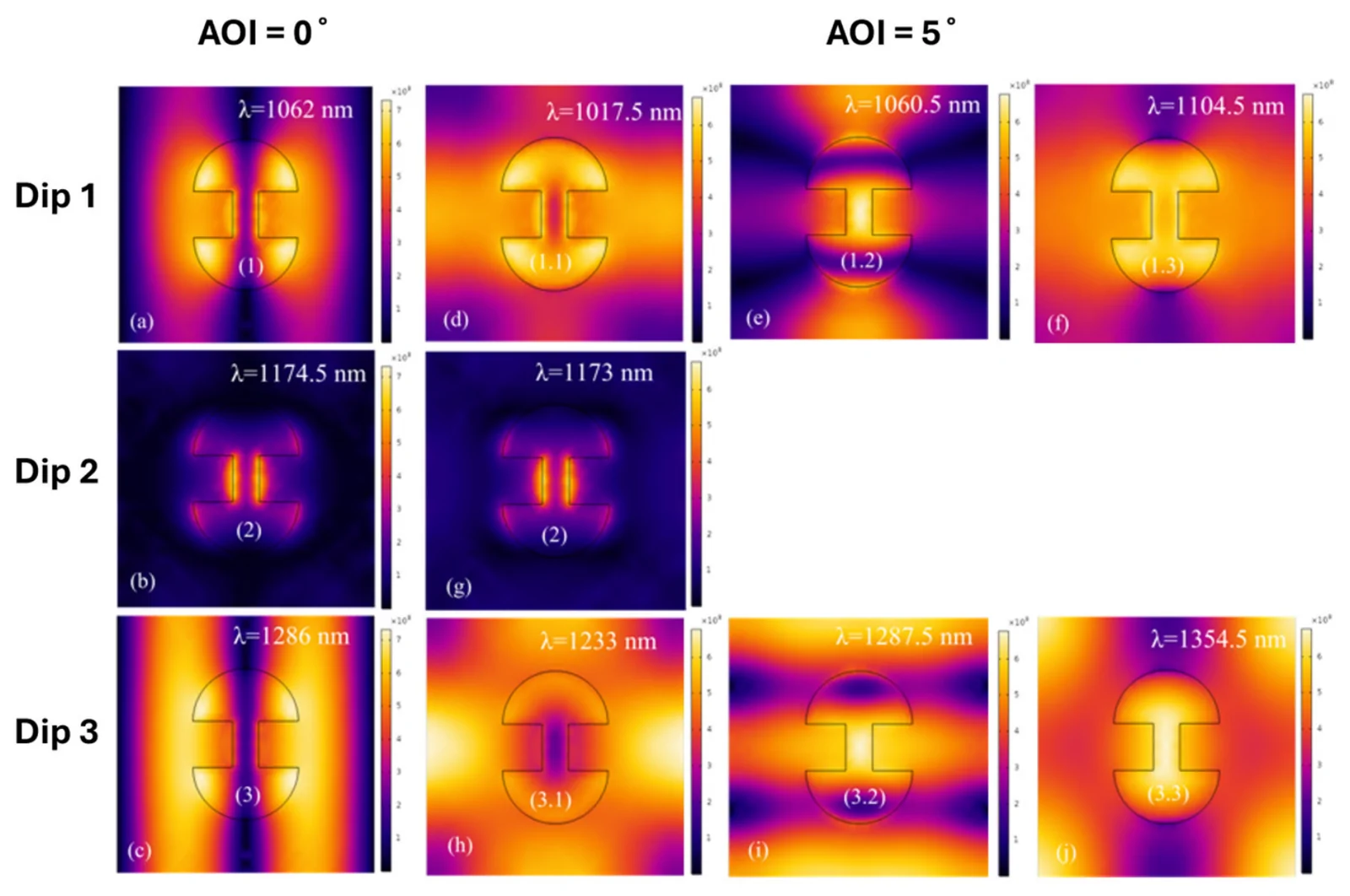
Electric-field distributions illustrating resonance evolution under *x*-oriented electric-field excitation: (**a**–**c**) correspond to Dip 1, Dip 2, and Dip 3 under normal incidence (AOI = 0°). Under oblique excitation (AOI = 3.5°), Dip 1 splits into three resonant branches, represented by (**d**–**f**). Dip 2 remains largely unchanged, as shown in panel (**g**), while Dip 3 splits into three distinct resonant branches, represented by (**h**–**j**).

The electric-field distributions shown in Figure 7 clarify the resonance formation mechanism under *x*-oriented electric-field excitation. At normal incidence (AOI = 0°), the metasurface supports three resonant modes at λ = 1062 nm, 1174.5 nm, and 1286 nm. The first resonance (Figure 7a) exhibits a relatively broad field distribution across the split-disk dimer, indicating weaker modal confinement and stronger radiative coupling. In contrast, the second resonance at λ = 1174.5 nm (Figure 7b) shows strong field localization near the inner dielectric boundaries and central coupling region. This enhanced confinement, together with coupling-induced interference between radiative and weakly radiative resonant pathways, suppresses radiative leakage and explains the ultranarrow linewidth and exceptionally high Q-factor observed for this mode. However, because only a relatively small fraction of the optical field extends into the surrounding analyte region, the interaction with refractive-index perturbations remains comparatively limited, resulting in lower sensitivity than that of the third resonance. The third resonance at λ = 1286 nm (Figure 7c) exhibits a more spatially extended field profile with stronger penetration into the surrounding medium, resulting in increased evanescent-field interaction with the analyte and consequently higher refractive-index sensitivity, albeit with a broader linewidth.

Under oblique excitation (AOI = 3.5°), the finite in-plane wave-vector component perturbs the excitation symmetry and causes the lateral resonances to split into multiple resonant branches. The original Dip 1 mode at λ = 1062 nm undergoes angular-induced splitting into three distinct branches at λ = 1017.5 nm, 1060.5 nm, and 1104.5 nm (Figure 7d–f). These split modes exhibit clear redistribution of electromagnetic confinement within the dielectric resonator, with the mode at λ = 1060.5 nm showing stronger localization near the central coupling region. The resonance at λ = 1173 nm (Figure 7g) preserves the highly localized field profile of the high-Q mode observed at normal incidence, indicating limited angular perturbation. Overall, these field distributions indicate that under *x*-polarized oblique excitation, Dip 1 and Dip 3 are significantly more sensitive to angular perturbation and undergo resonance splitting through modified modal coupling conditions, whereas Dip 2 preserves its strongly confined high-Q modal characteristics with only limited spectral perturbation.

## 5. Results and Discussion

Under normal-incidence excitation, the optimized metasurface exhibits a clear multi-resonant response for both polarization states, with three resonant dips appearing within the investigated wavelength range. The observed behavior is consistent with electromagnetic coupling between the two dielectric resonators mediated by the central connecting bar, giving rise to collective resonant states with distinct radiative characteristics. The central-bar-coupled split-disk dimer geometry governs the modal coupling conditions and spatial field distribution, leading to the excitation of multiple resonant modes at distinct wavelengths. The electric-field distributions presented in Figure 6 and Figure 7 further reveal that different resonances exhibit different degrees of electromagnetic confinement and evanescent-field penetration into the surrounding sensing medium, which directly influence their linewidth, Q-factor, and refractive-index sensitivity. The presence of multiple resonant modes is advantageous for biosensing because it enables simultaneous monitoring of several spectrally distinct sensing channels, allowing for performance comparison between resonances and providing greater flexibility in selecting the most suitable resonance according to the required balance between sensitivity and spectral resolution.

The results presented in Section 4.1, Section 4.2 and Section 4.3 further indicate that the resonant response is governed by collective hybridized modes supported by the complete central-bar-coupled split-disk dimer rather than by independent resonances of the individual semicylindrical elements. In particular, the central connecting bar acts as an electromagnetic coupling pathway that hybridizes the resonant modes of the two dielectric sections, producing coupled states with different radiative efficiencies. The ultranarrow Dip 2 is consistent with a resonant state exhibiting reduced radiative coupling. The observed narrow linewidth and high Q-factor suggest reduced radiative leakage and increased photon lifetime compared with the other resonant modes. The angular response further supports this interpretation, since the introduction of a finite in-plane wave vector under oblique incidence perturbs the excitation symmetry of the coupled modes, enabling previously overlapping resonant states to become spectrally distinguishable. The resulting resonance splitting and linewidth evolution are consistent with coupling between resonant pathways exhibiting different radiative efficiencies and qualitatively resemble Fano-like interference. Although the present work does not include the eigenmode or multipole analyses required for an unambiguous modal classification, the consistent correlation between resonance evolution, electric-field localization, polarization dependence, and symmetry-controlled resonance splitting provides a physically consistent interpretation of the observed high-Q resonances.

For *y*-polarized excitation, the three resonances exhibit narrow linewidths, with the second dip providing the most favorable sensing response because it simultaneously combines the highest Q-factor and refractive-index sensitivity. This combination of strong spectral selectivity and large resonance shift makes Dip 2 particularly suitable for high-resolution refractive-index sensing under this polarization.

A similar trend is observed for *x*-polarized excitation, where the second resonance exhibits exceptional spectral selectivity owing to its ultranarrow linewidth and *Q*-factor exceeding 2000. From a sensing perspective, the second and third dips provide the highest sensitivity values, equal to 830 nm/RIU and 860 nm/RIU, respectively. Therefore, while the third resonance exhibits the largest wavelength shift upon refractive-index variation, the second resonance offers the best compromise between sensitivity and spectral sharpness, resulting in superior overall sensing performance.

More broadly, high-Q resonances in metasurfaces have been recently pursued through several distinct design strategies. Bound states in the continuum (BIC) and their radiatively accessible quasi-BIC counterparts provide strong electromagnetic confinement and sharp Fano resonances [30]. Recent implementations include Brillouin-zone folding to sustain ultrahigh-Q behavior over broader regions of momentum space and improve robustness against structural disorder [31], centroid-symmetry-protected states and area-conserved guided-mode resonances in composite tetramer metasurfaces [32], intrinsic chiral BICs obtained through combined in-plane and out-of-plane symmetry breaking [33], and precision-controlled angular perturbations for reproducible quasi-BIC excitation [34]. High-Q magnetic-dipole quasi-BICs have also been exploited in ultrathin silicon metasurfaces to enhance nonlinear optical interactions [35]. Together, these studies show that symmetry control, collective lattice effects, guided-mode engineering, and controlled perturbations provide complementary routes to narrow-linewidth optical resonances.

The angular analysis shows that the metasurface response is not simply degraded when moving away from normal incidence, but rather evolves into additional split resonant modes whose behavior depends strongly on the incident polarization. This indicates that the in-plane wave-vector component introduced at oblique incidence modifies the symmetry of the excitation and allows for coupling to modes that are not equivalently accessed at AOI = 0°.

For *y*-polarized excitation, the most relevant effect is the splitting of Dip 2 into two narrower dips. This is particularly interesting because the angular variation does not broaden the original mode, but instead produces two sharper resonances with lower FWHM values than the normal-incidence resonance. From a sensing perspective, this behavior could be advantageous because the two split resonances preserve the same high SB of the original central dip, around 830 nm/RIU, while offering improved spectral sharpness. Therefore, oblique excitation under *y*-polarization can be used to increase the number of available sensing channels without compromising sensing performance.

For *x*-polarized excitation, the angular response mainly affects the two lateral resonances, each of which splits into three distinct dips. Although this leads to a more structured spectrum, the resulting split resonant modes remain well defined and generally narrower than the corresponding resonances at normal incidence. The sensing analysis confirms that these resonances retain high refractive-index sensitivity. In particular, the resonances derived from the second lateral dip show sensitivities of 870, 860, and 850 nm/RIU, slightly higher than the value obtained at normal incidence for the same spectral region.

To contextualize the sensing performance of the proposed metasurface, Table 2 compares representative refractive-index sensing metasurfaces operating in the visible and near-infrared spectral ranges, including all-dielectric, plasmonic, and hybrid metal–dielectric platforms. Among the all-dielectric designs, the missing wedge-shaped nanodisk metasurface, consisting of silicon nanodisks on a SiO_2_ substrate, operates at 925 nm and combines a high Q of 1202 with a sensitivity of 83 nm/RIU and a FOM of 92 RIU^−1^ [36]. Other all-dielectric configurations show improved sensing response: the single-block structure with nanogaps exhibits a resonance of around 1550 nm, a Q-factor of 1233, an S_B_ of 301 nm/RIU and an FOM = 239 RIU^−1^ [37], while the cylindrical nanodisk Huygens metasurface, based on amorphous silicon nanoantennas on a glass substrate, operates at 1217 nm with a sensitivity of 350 nm/RIU and FOM = 219 RIU^−1^ [38]. The best Q-factor and FOM among the all-dielectric platforms considered here are reported for the E-shaped nanorod metasurface, composed of mirror-symmetric silicon nanorods on a silica substrate, which resonates at 1139 nm and reaches Q = 7253, S_B_ = 430 nm/RIU, and FOM = 1720 RIU^−1^ [39]. However, these superior figures are achieved at the expense of a substantially more complex meta-atom geometry compared with the other all-dielectric configurations considered here. Overall, these results confirm the ability of dielectric metasurfaces to support narrow resonances and high spectral selectivity, although their sensitivity generally remains moderate compared with plasmonic or hybrid platforms.

In contrast, the plasmonic nano-rod sensor included in the comparison exhibits a much larger sensitivity of 1125 nm/RIU at 1398 nm, but with a lower FOM of 75 RIU^−1^ [40]. This highlights the typical trade-off of plasmonic sensing platforms, where strong field confinement and large resonance shifts are often accompanied by broader resonances due to metallic losses. Hybrid metal–dielectric metasurfaces provide an intermediate route by combining the strong field localization associated with metallic layers and the sharper resonant response of dielectric meta-atoms. In this class, a metasurface based on a square meta-atom over an Au reflector operating around 1550 nm achieves Q = 514, S_B_ = 1200 nm/RIU, and FOM = 324 RIU^−1^ [41]. A nanopillar-on-Au-reflector design shows a resonance around 700 nm, with Q = 2370 and sensitivity close to 500 nm/RIU [25], while a nanodisk-on-Au-reflector configuration operating around 1050 nm reaches a sensitivity of 325 nm/RIU [24]. Another hybrid design based on a dielectric patch on an Al metal plane operates at 1264 nm and reports Q = 120, S_B_ sensitivity = 840 nm/RIU, and FOM = 84 RIU^−1^ [26].

The proposed hybrid metasurface configurations demonstrate sensing performances that are highly competitive with, and in several cases superior to, the state-of-the-art platforms summarized in Table 2. In particular, the *x*-oriented incident-light configuration exhibits outstanding performance for the second resonance dip, which combines an ultranarrow linewidth (FWHM below 1 nm) with high bulk sensitivity, resulting in an FOM of 1431 RIU^−1^. This confirms that the proposed metasurface provides an effective compromise between high sensitivity and narrow resonance linewidth, as reflected by its particularly high FOM, while preserving multi-resonant operation and therefore enabling multiple potential sensing channels within a relatively simple hybrid geometry. This performance surpasses most previously reported plasmonic and hybrid metasurfaces and approaches the values reported only for considerably more complex all-dielectric architectures such as the E-shaped nanorod metasurface [20]. Importantly, the proposed structure achieves these performances using a comparatively simple geometry composed of two dielectric semicylinders connected by a central bar on a metallic film, thereby combining high-Q resonances, strong refractive-index sensitivity, and geometrical simplicity within a compact sensing platform.

Although not serving as direct benchmarks, recently reported metasurfaces operating in the THz regime can still provide valuable comparative context due to their conceptual similarity in resonant meta-atom engineering and frequency-domain sensing methodologies. A BDS triple-PIT metasurface combining open-rectangular-ring and U-shaped resonators was reported in the 0.5–3.5 THz range, with four resonant dips between 0.847 and 2.957 THz and a maximum sensitivity of 1.39 THz/RIU with FOM = 114.46 RIU^−1^ [42]. Another BDS-based design, closer in spirit to multi-band absorber-based sensing, employed stacked double-ring meta-atoms and showed four narrow absorption peaks between 6.836 and 9.362 THz, Q-factors up to 227.9, maximum sensitivity of 871 GHz/RIU and FOM = 18.9 RIU^−1^ [43]. Borophene has also been explored in a PIT-like metasurface composed of two strips and a central rectangle, operating over 120–215 THz and reaching sensitivities of 46.26 and 56.47 THz/RIU with FOM up to 55.21 RIU^−1^ [44].

From a practical implementation perspective, the proposed hybrid metasurface remains compatible with established nanofabrication technologies. The continuous Au layer can be deposited using electron-beam evaporation or sputtering, followed by Si_3_N_4_ thin-film deposition using plasma-enhanced chemical vapor deposition. The dielectric meta-atoms can subsequently be patterned using high-resolution electron-beam lithography combined with reactive ion etching [45,46]. Although the central connecting bar width is only 100 nm, such dimensions remain within the capability of current nanofabrication processes. In practice, fabrication imperfections such as corner rounding, sidewall angle deviations, surface roughness, or small dimensional variations may introduce moderate resonance shifts and linewidth broadening, potentially reducing the Q-factor because of increased scattering losses. However, since the sensing mechanism relies on hybrid dielectric–plasmonic resonant coupling rather than extremely sharp geometrical singularities, moderate fabrication imperfections are not expected to significantly alter the fundamental sensing mechanism of the proposed structure.

Although narrow resonance linewidth generally improves theoretical sensing performance by enhancing spectral resolution and increasing the FOM, the practical detectability of narrow resonance features depends strongly on the characteristics of the interrogation system used during experimental implementation. Modern high-resolution optical characterization platforms, including tunable laser interrogation systems and high-precision optical spectrum analyzers, are capable of reliably resolving sub-nanometer spectral features [47]. However, under lower-resolution or noise-limited measurement conditions, very narrow resonance dips may introduce additional challenges in wavelength tracking and signal stability. Therefore, optimal experimental performance requires an appropriate balance among resonance linewidth, resonance contrast, and detection system sensitivity.

In addition to resonance linewidth, the reflectance contrast of the resonance dip also plays an important role in practical sensing performance. Although the second resonance exhibits the highest Q-factor owing to its significantly reduced linewidth, its relatively reduced reflectance depth may decrease signal contrast during experimental interrogation. In practical measurements, shallow resonance dips can become more sensitive to detector noise, intensity fluctuations, and baseline instability, potentially reducing the signal-to-noise ratio during wavelength tracking. However, modern optical interrogation systems employing high-stability light sources and sensitive photodetection schemes can significantly mitigate these limitations. Therefore, practical sensor performance requires an appropriate balance among resonance linewidth, resonance contrast, and detection system sensitivity.

From a practical biosensing perspective, the reported refractive-index sensitivities indicate that the proposed metasurface is responsive to small dielectric perturbations of the type that may be generated by biomolecular interactions occurring near the sensor surface. Importantly, the simultaneous combination of relatively high refractive-index sensitivity with ultranarrow resonance linewidths enables improved wavelength-shift resolution, which is particularly critical for reliable label-free detection of low-concentration analytes. Furthermore, the availability of multiple resonant channels together with polarization-dependent spectral behavior may provide additional flexibility for future multi-parameter or multi-analyte optical biosensing implementations.

## 6. Conclusions

This work demonstrates a hybrid MSA based on a central-bar-coupled split-disk dimer for high-resolution refractive-index sensing in the near-infrared spectral region. By combining a Si_3_N_4_ dielectric resonator with a continuous Au plasmonic layer, the proposed design supports multiple sharp resonant modes within the 1000–1400 nm wavelength range. The optimized structure exhibits strong spectral selectivity, with FWHM values as low as 0.58 nm and *Q*-factors exceeding 2000 under *x*-oriented electric-field excitation. The refractive-index analysis confirms strong sensing capability, with bulk sensitivities reaching 860 nm/RIU under normal incidence and up to 870 nm/RIU under oblique excitation.

The angular investigation further reveals that small deviations from normal incidence do not simply deteriorate the optical response, but instead induce additional polarization-dependent resonant modes. These angularly induced resonances preserve narrow linewidths and high refractive-index sensitivity, suggesting that controlled oblique excitation may provide additional spectral degrees of freedom for multi-channel sensing and improved spectral discrimination. Electric-field analysis further confirms strong electromagnetic localization near the dielectric boundaries, central coupling region, and metal-dielectric interface, supporting efficient interaction between the confined optical fields and the surrounding analyte.

The proposed metasurface therefore represents a compact and spectrally selective platform for high-performance biosensing in the near-infrared regime. Future work should focus on experimental realization, fabrication-tolerance analysis, and integration with microfluidic platforms to assess practical sensing performance under realistic operating conditions. In addition, surface functionalization strategies and analyte-specific investigations will be essential to extend the present refractive-index sensing framework toward selective biochemical and biomedical detection.

## Figures and Tables

**Table 1:** Comparison of the performances for both incident electric field orientations under normal incidence (AOI = 0°) and oblique incidence (AOI ≠ 0°).

E-Field Orientation	Resonance Dips		Resonance Wavelength (nm)	FWHM (nm)	S_B_ (nm/RIU)
*y*-Oriented E AOI = 0°	Dip 1		1070	1.93	550
	Dip 2		1175.5	1.19	830
	Dip 3		1310	1.85	800
*x*-Oriented E AOI = 0°	Dip 1		1062	3.42	550
	Dip 2		1174.5	0.58	830
	Dip 3		1286.5	2.78	860
*y*-Oriented E AOI = 5°	Dip 1		1068.5	1.82	530
	Dip 2	Dip 2.1	1103	0.86	830
		Dip 2.2	1248	0.65	830
	Dip 3		1308	1.73	800
*x*-Oriented E AOI = 3.5°	Dip 1	Dip 1.1	1017.5	2.88	580
		Dip 1.2	1060.5	1.03	560
		Dip 1.3	1104.5	1.53	530
	Dip 2		1173	0.72	830
	Dip 3	Dip 3.1	1233	1.74	870
		Dip 3.2	1287.5	1.78	860
		Dip 3.3	1354.5	1.33	850

**Table 2:** Comparison of the performance of the proposed MSA with previously reported works.

Technology	MA Shape	Resonance Wavelength (nm)	Q-Factor	Sensitivity S_B_ (nm/RIU)	FOM S_B_/FWHM (RIU^−1^)	Ref.
All dielectric	Missing wedge-shaped nanodisks	925	1202	83	92	[36]
All dielectric	Single block with nanogaps	≈1550	1233	301	239	[37]
All dielectric	Cylindrical nanodisk	1217	-	350	219	[38]
All dielectric	E-shaped nanorods	1139	7253	430	1720	[39]
Plasmonic	Nano-rod	1398	-	1125	75	[40]
Hybrid	Square MA over Au reflector	≈1550	514	1200	324	[41]
Hybrid	Nanopillar on Au reflector	≈700	2370	≈500	-	[25]
Hybrid	Nanodisk on Au reflector	≈1050	-	325	-	[24]
Hybrid	Dielectric patch on Al metal plane	1264	120	840	84	[26]
Proposed configuration for *x*-oriented incident light	Two dielectric semicylinders connected by a central bar on Au film	1062 1174.5 1286.5	311 2007 463	550 830 860	160 1431 309	This work
Proposed configuration for *y*-oriented incident light	Two dielectric semicylinders connected by a central bar on Au film	1070 1175.5 1310	555 990 710	550 830 800	285 697 432	This work

## Data Availability

The data will be made available upon reasonable request to the corresponding authors.

## Supplementary Materials

The following supporting information can be downloaded at: https://www.mdpi.com/article/10.3390/bios16080439/s1, Note S1: Physical Interpretation of the Resonance Formation Mechanism.

## References

[1] Allayarov I., Aita V., Roth D.J., van Casteren B., Bykov A.Y., Evlyukhin A.B., Zayats A.V., Calà Lesina A. (2025). Strong Coupling of Collective Optical Resonances in Dielectric Metasurfaces. Light Sci. Appl..

[2] Abdollahramezani S., Hemmatyar O., Taghinejad M., Taghinejad H., Kiarashinejad Y., Zandehshahvar M., Fan T., Deshmukh S., Eftekhar A.A., Cai W. (2021). Dynamic Hybrid Metasurfaces. Nano Lett..

[3] Butt M.A. (2026). Flatland Metasurfaces for Optical Gas Sensing. Sensors.

[4] Avraham M., Nemirovsky Y. (2025). Multiphysics Optical–Thermal and Mechanical Modeling of a CMOS-SOI-MEMS Infrared Sensor with Metasurface Absorber. Sensors.

[5] Panciera C., Brunetti G., Ciminelli C. (2026). Numerical Investigation of IgG Sensing through Nanoscale Topological-Assisted Metasurface for Liquid Biopsy. Nanotechnol. Rev..

[6] Dell’Olio F., Angulo Barrios C. (2025). On-Chip Biosensing and Gas Sensing by Photonic Slot Waveguides: A Review. IEEE Sens. J..

[7] Ibarlucea B., Fernández-Sánchez C., Demming S., Büttgenbach S., Llobera A. (2011). Selective Functionalisation of PDMS-Based Photonic Lab on a Chip for Biosensing. Analyst.

[8] Altug H., Oh S.-H., Maier S.A., Homola J. (2022). Advances and Applications of Nanophotonic Biosensors. Nat. Nanotechnol..

[9] Rabbani A., Rudacille R., Hasegawa K. (2024). Local Refractive Index Sensitivity of Localized Surface Plasmon Resonance Biosensors. J. Phys. Chem. C.

[10] Agrahari R., Dwivedi S., Jain P.K., Mahto M. (2023). High Sensitive Metasurface Absorber for Refractive Index Sensing. IEEE Trans. Nanotechnol..

[11] Guo W., Liu Y., Han T. (2016). Ultra-Broadband Infrared Metasurface Absorber. Opt. Express.

[12] Butt M.A., Kazansky N.L. (2020). Narrowband Perfect Metasurface Absorber Based on Impedance Matching. Photonics Lett. Pol..

[13] Iwanaga M., Hu Q., Tang Y. (2025). Metasurface Biosensors: Status and Prospects. Appl. Phys. Rev..

[14] Chen Z., Huang J., Tan Q., Xiao G., Sun T., Zhang F., Idris A., Zou Q., Li H., Lu G.-W. (2026). High-Sensitivity Optical Sensor Driven by the High-Q Quasi-Bound States in the Continuum of an Asymmetric Bow-Tie Metasurface. Photonics.

[15] Li H., Shi Z., Zhang H., Qiu S., Zhou Z.-K. (2025). Hybrid Metasurface Based on Si_3_N_4_ Nanopillar for Optical Sensing with Dual Channels. ACS Appl. Nano Mater..

[16] Abdollahramezani S., Taghinejad H., Fan T., Marzban M.R., Eftekhar A.A., Adibi A. (2022). Reconfigurable Multifunctional Metasurfaces Employing Hybrid Phase-Change Plasmonic Architecture. Nanophotonics.

[17] Butt M.A., Khonina S.N., Kazanskiy N.L., Piramidowicz R. (2022). Hybrid Metasurface Perfect Absorbers for Temperature and Biosensing Applications. Opt. Mater..

[18] Luo M., Zhou Y., Zhao X., Guo Z., Li Y., Wang Q., Liu J., Luo W., Shi Y., Liu A.Q. (2024). High-Sensitivity Optical Sensors Empowered by Quasi-Bound States in the Continuum in a Hybrid Metal–Dielectric Metasurface. ACS Nano.

[19] Sun K., Li J., Ge L., Zhong K., Wang Y., Xu D., Yang X., Fu W., Yao J. (2022). Graphene-Enhanced Hybrid Terahertz Metasurface Sensor for Ultrasensitive Nortriptyline Sensing and Detection. Opt. Express.

[20] Wu X., Chen J., Yang J. (2025). Optically Transparent and Wideband Absorption-Tunable Metasurface Based on Patterned Graphene. Opt. Mater. Express OME.

[21] Du Z., Zhou R., Luo S., Zhao D., Long W., Ling Q., Yu Z., Chen D. (2023). Tunable Multi-Band Absorbers Based on Graphene Metasurfaces for Infrared Sensing and Switching. Opt. Commun..

[22] García García B., Fernández-Manteca M.G., Zografopoulos D.C., Gómez-Galdós C., Ocampo-Sosa A.A., Rodríguez-Cobo L., Algorri J.F., Cobo A. (2025). Plasmonic and Dielectric Metasurfaces for Enhanced Spectroscopic Techniques. Biosensors.

[23] Meng J., Balendhran S., Sabri Y., Bhargava S.K., Crozier K.B. (2024). Smart Mid-Infrared Metasurface Microspectrometer Gas Sensing System. Microsyst. Nanoeng..

[24] Rifat A.A., Rahmani M., Xu L., Miroshnichenko A.E. (2018). Hybrid Metasurface Based Tunable Near-Perfect Absorber and Plasmonic Sensor. Materials.

[25] Qiu S., Zhang H., Shi Z., Li H., Zhou Z.-K. (2023). Ultrasensitive Refractive Index Sensing Based on Hybrid High-Q Metasurfaces. J. Phys. Chem. C.

[26] Lu X., Zhang T., Wan R., Xu Y., Zhao C., Guo S. (2018). Numerical Investigation of Narrowband Infrared Absorber and Sensor Based on Dielectric-Metal Metasurface. Opt. Express.

[27] Bosomtwi D., Babicheva V.E. (2023). Beyond Conventional Sensing: Hybrid Plasmonic Metasurfaces and Bound States in the Continuum. Nanomaterials.

[28] Chen X., Leng R., Liu K., Guo C., Zhu Z., Qin S., Zhang J. (2023). High Quality Factor Resonant Metasurface with Etchless Lithium Niobate. Opt. Laser Technol..

[29] TalebiFard S., Schmidt S., Shi W., Wu W., Jaeger N.A.F., Kwok E., Ratner D.M., Chrostowski L. (2017). Optimized Sensitivity of Silicon-on-Insulator (SOI) Strip Waveguide Resonator Sensor. Biomed. Opt. Express BOE.

[30] Kang M., Liu T., Chan C.T., Xiao M. (2023). Applications of Bound States in the Continuum in Photonics. Nat. Rev. Phys..

[31] Wang W., Srivastava Y.K., Tan T.C., Wang Z., Singh R. (2023). Brillouin Zone Folding Driven Bound States in the Continuum. Nat. Commun..

[32] Zhou C., Jin R., He H., Huang J., Li G., Huang L. (2026). Robust Ultrahigh-Q Resonances in Tetramer Metasurfaces through Centroid Symmetry Protection and Area Conservation. Light Sci. Appl..

[33] Chen Y., Deng H., Sha X., Chen W., Wang R., Chen Y.-H., Wu D., Chu J., Kivshar Y.S., Xiao S. (2023). Observation of Intrinsic Chiral Bound States in the Continuum. Nature.

[34] Zhou C., Zhou M., Fu Z., He H., Deng Z.-L., Xiang H., Chen X., Lu W., Li G., Han D. (2025). Ultrahigh-Q Quasi-BICs via Precision-Controlled Asymmetry in Dielectric Metasurfaces. Nano Lett..

[35] Xu L., Zangeneh Kamali K., Huang L., Rahmani M., Smirnov A., Camacho-Morales R., Ma Y., Zhang G., Woolley M., Neshev D. (2019). Dynamic Nonlinear Image Tuning through Magnetic Dipole Quasi-BIC Ultrathin Resonators. Adv. Sci..

[36] Zhao Y., Geng Q., Liu J., Geng Z. (2024). Sensing Characteristic Analysis of All-Dielectric Metasurfaces Based on Fano Resonance in Near-Infrared Regime. Photonics.

[37] Watanabe K., Iwanaga M. (2023). Nanogap Enhancement of the Refractometric Sensitivity at Quasi-Bound States in the Continuum in All-Dielectric Metasurfaces. Nanophotonics.

[38] Ollanik A.J., Oguntoye I.O., Hartfield G.Z., Escarra M.D. (2019). Highly Sensitive, Affordable, and Adaptable Refractive Index Sensing with Silicon-Based Dielectric Metasurfaces. Adv. Mater. Technol..

[39] Li H., Xing J., Shi Y., Yu S., Zhao T. (2023). Performance Analysis and Optimization of High Q-Factor Toroidal Resonance Refractive Index Sensor Based on All-Dielectric Metasurface. Opt. Laser Technol..

[40] Baranzadeh F., Nozhat N. (2019). Tunable Metasurface Refractive Index Plasmonic Nano-Sensor Utilizing an ITO Thin Layer in the near-Infrared Region. Appl. Opt. AO.

[41] Butt M.A. (2025). Ultra-Sensitive Refractive Index Sensing Enabled by High-Q Hybrid Plasmonic Metasurface Absorbers. Opt. Laser Technol..

[42] Chen F., Xu B., Liu B. (2026). Multiple Modulator and Sensor in a Bulk Dirac Semimetal Metamaterial Based on Triple Plasmon-Induced Transparency. Micro Nanostructures.

[43] Li L., Chen F. (2025). Tunable Four-Band Metamaterial Absorber and Sensor Based on a Stacking Double-Ring Dirac Semimetal Structure Design. Phys. Lett. A.

[44] Gao W., Chen F., Yang W. (2025). Modulator and Sensor Based on In-Plane Mode Weak Coupling in Borophene Metamaterial. Photonics Nanostructures-Fundam. Appl..

[45] Vieu C., Carcenac F., Pépin A., Chen Y., Mejias M., Lebib A., Manin-Ferlazzo L., Couraud L., Launois H. (2000). Electron Beam Lithography: Resolution Limits and Applications. Appl. Surf. Sci..

[46] Dirdal C.A., Jensen G.U., Angelskår H., Thrane P.C.V., Gjessing J., Ordnung D.A. (2020). Towards High-Throughput Large-Area Metalens Fabrication Using UV-Nanoimprint Lithography and Bosch Deep Reactive Ion Etching. Opt. Express.

[47] Luo Y.-H., Shi B., Sun W., Chen R., Huang S., Wang Z., Long J., Shen C., Ye Z., Guo H. (2024). A Wideband, High-Resolution Vector Spectrum Analyzer for Integrated Photonics. Light Sci. Appl..

