# Supplementary material for: Ultra-Broadband Metasurface Absorber Enabled by a Central-Bar-Coupled Split-Disk Dimer
Source: Biosensors (Basel). 2026 Aug 14;16(8):439. doi: 10.3390/bios16080439 (PMC13511334; doi:10.3390/bios16080439)
Supplement: Supplementary file 1 [file biosensors-16-00439-s001.zip › biosensors-4468612-supplementary.pdf]

Supplementary Note S1. Physical Interpretation of the Resonance Formation Mechanism

The resonant response of the proposed metasurface should be interpreted as the collective electromagnetic behavior of the complete central-bar-coupled split-disk dimer rather than as the superposition of independent resonances supported by the individual semicylindrical dielectric elements. The central connecting bar provides an electromagnetic coupling pathway between the two Si_3N_4 sections, allowing the resonant fields to extend across the entire dielectric meta-atom. Consequently, the optical response is consistent with collective resonant states whose spatial field distributions and radiative characteristics are governed by the coupled geometry of the dielectric resonator rather than by isolated resonances of the individual semicylinders.

The continuous Au layer performs a complementary electromagnetic function by modifying the optical boundary conditions beneath the dielectric resonator. Unlike conventional nanoparticle-on-a-mirror (NPoM) architectures, where localized plasmonic cavity modes are supported by a metallic nanoparticle separated from a metallic mirror by a nanometer-scale gap, the present structure consists of dielectric resonators fabricated on a continuous Au reflector. The role of the Au layer is therefore primarily to suppress optical transmission, increase electromagnetic confinement near the dielectric–metal interface, and strengthen the interaction between the evanescent field and the surrounding sensing medium. Consequently, the observed resonances should not be interpreted as conventional localized plasmonic cavity modes but rather as resonant states supported by the coupled dielectric resonator in the presence of a metallic reflecting boundary.

The electric-field distributions presented in Figures 6 and 7 of the main manuscript provide further physical insight into the observed resonant behavior. The first resonance exhibits a comparatively broad field distribution throughout the dielectric resonator, indicating stronger interaction with free-space radiation and consequently a broader spectral linewidth. In contrast, Dip 2 exhibits the strongest field localization near the central coupling region and the inner dielectric boundaries while simultaneously producing the narrowest linewidth and the highest quality factor. This correlation is consistent with a resonant state exhibiting reduced radiative coupling, allowing electromagnetic energy to remain confined within the resonator for a longer period of time before being radiated away, thereby increasing the effective photon lifetime and producing the experimentally observed ultranarrow resonance. The third resonance exhibits a more spatially extended evanescent field penetrating further into the surrounding analyte region, explaining its comparatively larger refractive-index sensitivity despite its broader linewidth.

The angular response provides additional evidence supporting this physical interpretation. Under normal incidence, the symmetry of the excitation restricts the coupling conditions of the collective resonant states, causing several resonances to remain spectrally overlapped or only weakly distinguishable. When the incidence angle becomes nonzero, the finite in-plane wave-vector perturbs the excitation symmetry and modifies the phase relationship between the coupled resonant pathways. Consequently, resonances that are nearly overlapped under normal incidence evolve into spectrally distinguishable branches, producing the experimentally observed resonance splitting. The accompanying linewidth reduction observed for several of these split resonances further suggests that symmetry perturbation modifies the radiative coupling efficiency of the corresponding resonant states.

Taken together, the combined evidence provided by the reflection spectra, electric-field distributions, polarization dependence, and angular evolution consistently supports the interpretation that the observed high-Q resonances originate from electromagnetic coupling between the two dielectric sections mediated by the central connecting bar in the presence of the

continuous Au reflector. The observed resonance evolution is therefore consistent with coupling-induced Fano-like interference between resonant pathways exhibiting different radiative efficiencies. However, the present work does not attempt an explicit modal classification through eigenmode analysis, multipole decomposition, or Fano fitting. Such techniques provide valuable tools for rigorous modal identification but are beyond the scope of the present study, whose primary objective is the design, optimization, and sensing performance of the proposed hybrid metasurface biosensor. The interpretation presented here is therefore intentionally based on the consistent correlation among the spectral response, field localization, polarization dependence, and angular evolution obtained from the numerical simulations.
